# Investor Sentiment and Stock Returns During the COVID-19 Pandemic

**DOI:** 10.3389/fpsyg.2021.708537

**Published:** 2021-07-20

**Authors:** Baozhen Jiang, Haojie Zhu, Jinhua Zhang, Cheng Yan, Rui Shen

**Affiliations:** ^1^School of Economics, Zhejiang University of Technology, Zhejiang, China; ^2^School of Applied Science and Technology, Hainan University, Haikou, China

**Keywords:** Baidu index, google trends, investor sentiment, epidemic stocks, forecast

## Abstract

In this paper, we regard the Baidu index as an indicator of investors' attention to China's epidemic stocks. We believe that when seeking information to guide investment decisions, investor sentiment is usually affected by the information provided by the Baidu search engine, which may cause stock prices to fluctuate. Therefore, we constructed a GARCH extended model including the Baidu index to predict the return of epidemic stocks and compared it with the benchmark model. The empirical research in this paper finds that the forecast model including the Baidu index is significantly better than the benchmark model. This has important reference value both for investors in predicting stock trends and for the government's formulation of policies to prevent excessive stock market volatility.

## Introduction

Information has become one of the most valuable assets in the financial market. A growing body of literature shows that there is a complex correlation between information and financial market fluctuations. However, due to data limitations, most of the previous research has focused on the exploration of the role of micro-enterprise financial information. Many studies have also theoretically explored the importance of information to financial markets. With the popularization of mobile Internet, the emergence of big data information has completely changed the production, intermediary, dissemination, and consumption of information in the financial industry. Investors usually use enhanced information searches to cope with uncertainty and assist decision-making. The main advantages of using Internet searches are that they are free, wide-ranging, and timeliness.

The use of information obtained from search engines for stock market return forecasts is gradually gaining attention. The availability of big data brought about by search engines reinforces this trend. These data can usually provide investors with real-time information, thereby enhancing the scientific nature of investment decisions. In view of the gradual popularity of the mobile Internet, it has become a standard practice for most investors to seek online information before making an investment decision. Investors leave behind data about what they are looking for every time they use a search engine. If these retrieved data are analyzed systematically, investor sentiment in the real world can be effectively tracked. Therefore, the analysis of stock market dynamics usually depends on the availability of online information. Among them, the use of Google search data to predict epidemics, unemployment rates, and epidemiology is widely known.

Although Google ranks first in the global search engine market, it is unavailable to Chinese users. In China, investors usually use the Baidu search engine to retrieve data. This search engine's market share ranks first in China. Therefore, it is reasonable to believe that the data provided by Baidu searches can reliably reflect the attention of Chinese investors. The Baidu Index is a tool similar to Google Trends, launched by Baidu to reflect the intensity and interest of users in searching for specific keywords. Unlike Google Trends' weekly data, the Baidu Index can obtain daily user retrieval data, but there is a fee to download this data. In the current context in which the COVID-19 epidemic has not yet been effectively controlled, the availability of such high-frequency data will not only assist consumers in making investment decisions, but also help government departments to monitor epidemic trends in real time, contributing to efforts to control the epidemic.

In the raging situation of the COVID-19 epidemic, most existing studies use Google Trends to analyze the epidemic's impact, predict its trends, determine the users' needs for epidemic prevention materials and information, and monitor epidemiological trends. There are also some studies using Google Trends to examine the impact of COVID-19 on the macro economy and the stock market (Szczygielski et al., 2021). However, studies using the Baidu index to examine the impact of investor sentiment on the returns of epidemic stocks are still rare. Although COVID-19 has dealt a huge blow to the tourism, hotel, and aviation industries, epidemic stocks such as anti-epidemic supplies and vaccines have benefited from this public health emergency (Wu et al., 2020). Using the Baidu index to proxy the information needs of investors based on Internet search volume and analyzing the impact of investor sentiment on specific stocks of the epidemic will not only help us to analyze the public's demand for epidemic materials and information, but to engage in real-time monitoring of the epidemic's trends and to improve the government's ability to respond and help in the economic recovery.

## Literature Review

In recent years, some scholars have used Google Trends to study epidemics and disease spread. There are also some studies that predict financial markets based on Google Trends data, but the conclusions are inconsistent.

Yung and Nafar (2017) have investigated the impact of retail investor attention to the expected returns of real estate investment trust funds. The study found that the higher the interest of retail investors as measured by the Google Trends search volume index, the higher the expected return of real estate investment trusts. Swamy et al. (2019) use data from S&P BSE 500 companies listed on the Indian Stock Exchange from 2012 to 2017 to examine the effectiveness of investor attention as measured by the Google search volume index in predicting stock returns. The results of the study show that a higher Google search volume index can predict positive and significant earnings in the following 1–2 weeks. Higher quantiles of GSVI will produce higher excess returns.

Research by Vlastakis and Markellos (2012) shows that the demand for information is positively correlated with the volatility and trading volume of 30 major stocks traded on the New York Stock Exchange and Nasdaq. Takeda and Wakao (2014) also argue that the increase in search activity is related to the increase in trading activity, but the possibility that the increase in trading volume will cause the stock price to rise is not high. Da et al. (2011) found that the increase in the Search Volume Index of Russell's 3000 shares indicates that the stock price will rise in the next 2 weeks, and the price will eventually reverse within a year. The Search Volume Index has also brought large first-day earnings and long-term underperformance to many IPO stocks. Nguyen et al. (2019) even believe that the increase in Google search volume in the Philippines, Thailand, and Vietnam had a significant negative impact on stock earnings.

Preis et al. (2010) investigate the correlation between company name search volume and rate of return, but they did not find a significant correlation between the two. Instead, they found strong evidence that Google search data can be used to predict transaction volume. Kim et al. (2019) study the impact of Google searches on Norwegian stock market activity. They also find that Google searches have no correlation to abnormal returns over the same period or in the future. Fang et al. (2020) used Baidu keyword search volume data to improve the volatility prediction of the Chinese stock market. (Gozgor et al., 2019; Jiang et al., 2020; Liu et al., 2020; Fang et al., 2021a,b; Wu et al., 2021) discussed the impact of economic uncertainty, complexity, COVID-19 and globalization on the economy and financial markets.

## Variable Description and Statistical Description

In view of the data availability and habits of Chinese investors using search engines, we use the Baidu index to analyze investors' attention to information about the epidemic. For keywords related to the epidemic, we have selected representative terms such as “N95 masks,” “Wuhan epidemic,” “latest news about COVID-19,” “medical masks,” etc. We use N95, Wuhan, COVID-19, and Mask to represent the above variables, respectively. All keyword data are taken with natural logarithmic values. We selected Walvax, a listed company, as the research object for the stock return data of the epidemic. Walvax is a modern biopharmaceutical enterprise specializing in the research and development, and the production and sales of biological drugs such as vaccines and blood products in China. It is a nationally recognized high-tech enterprise and a national enterprise technology center. The company was listed on the Growth Enterprise Market of the Shenzhen Stock Exchange of China in November 2010. Its stock code is 300142. We use the following formula to calculate stock returns:

(1) $$Return_{i,t}=\frac{\left(\mathrm{Closing}\;price_{i,t}-\mathrm{Closing}\;price_{i,t-1}\right)}{\mathrm{Closing}\;price_{i,t-1}}$$

In the above formula, Closing *price*_*i,t*_ represents the closing price of stock *i* on day *t*. All stock return data are multiplied by 100.

Before exiting the Chinese market in 2010, Google ranked first in the Chinese search engine market. Since then, Baidu's market share has continued to rise from 60.2% in 2010 to 72.4% in May 2021 (Statcounter, 2021). In view of Baidu's leading position in the current Chinese search engine market, we choose Baidu Index instead of Google Trends as the source of search data to obtain investor sentiment (http://zhishu.baidu.com/).

The statistical description of the main variables is shown in Table 1. From the perspective of stock return, the maximum value is 12.423, which was obtained on February 10, 2021, and the minimum value is −19.996, which was obtained on December 7, 2020. Among all search keywords, “latest news about COVID-19” has the largest average value, because investors will continue to pay attention to the progress of the epidemic. The keyword “medical masks” has the smallest mean value, because investors' attention to medical masks has declined as the epidemic in China was quickly brought under control.

**Table 1:** Statistical description of the main variables.

	**Return**	**N95**	**Wuhan**	**COVID-19**	**Mask**
Mean	0.016	6.424	7.617	10.058	5.930
Median	0.096	6.417	7.617	10.041	5.872
Maximum	12.423	7.272	9.526	11.439	7.403
Minimum	−19.996	5.429	6.758	9.141	4.997
Std. Dev.	4.129	0.325	0.475	0.486	0.438
Skewness	−0.370	−0.500	0.744	0.625	0.641
Kurtosis	5.895	3.891	4.352	3.273	4.271
Jarque-Bera	68.468	13.739	30.971	12.536	25.008
Probability	0	0.001039	0	0.001896	0.000004
Sum	2.910	1182.072	1401.443	1850.660	1091.151
Sum Sq. Dev.	3119.236	19.308	41.262	43.163	35.151
Observations	184	184	184	184	184

## Empirical Research

Before empirical research, we need to test the stationarity of the data. We use ADF and PP tests to investigate the stationarity of the data, and the test results are shown in Table 2. The test results show that there is a unit root in the time series of stock closing prices. But the stock market return rate has overcome this problem because of the differential. Table 2 shows that both Return and N95 variables passed the 1% significance test. The remaining variables have passed the 10% statistical test. This shows that all time series are stationary.

**Table 2:** Data stationarity test.

	**ADF**	**PP**
Return	−16.31137^***^	−16.31657^***^
N95	−3.686776^***^	−3.752247^***^
Wuhan	−3.711559^***^	−3.418142^**^
COVID-19	−2.856401^*^	−2.974697^**^
Mask	−3.359155^**^	−3.365990^**^

***, **, and * *represent statistical significance at 1, 5, and 10% levels, respectively*.

The forecast error of financial time series usually has heteroscedasticity. The size of the residual is related to the most recent residual value. Ignoring the effects of ARCH may lead to reduced effectiveness. We use the ARCH LM method to test the ARCH effect.

The null hypothesis of the ARCH LM test is: there is no ARCH effect up to the order in the residual sequence, and the following regression is required:

(2) $${\hat{u}}_{t}^{2}=\alpha_{0}+(\sum_{s=1}^{p}\alpha_{s}{\overset{⌢}{u}}_{t-s}^{2})+\varepsilon_{t}$$

In formula (2), û_*t*_ represents the residual. Under the null hypothesis, the exact finite sample distribution of the F statistic is unknown. LM test generally follows the χ^2^(p) distribution gradually.

The test results are shown in Table 3. The LM test results show that the null hypothesis is rejected at a significance level of 10%, indicating that the residual sequence of formula (2) has an ARCH effect. In addition, from the perspective of the fluctuation of the residual square, it also has the characteristics of time varying and clustering, which is suitable for modeling with a GARCH model.

**Table 3:** LM test results.

Breusch-Godfrey Serial Correlation LM Test:			
Null hypothesis: No serial correlation at up to 1 lag			
F-statistic	3.486551	Prob. F(1,245)	0.0631
Obs*R-squared	3.535849	Prob. Chi-Square (1)	0.0601

Since many asset prices are conditionally heteroscedastic, GARCH is widely used in the financial industry. We first estimate the GARCH(*p,q*) model, and then select the appropriate number of lag periods based on the AIC value of the variance equation. The estimation results show that GARCH(1,1) can better describe this process. Therefore, we choose the following benchmark model:

(3) $$\sigma^{2}=\omega +\alpha_{1}u_{t-1}^{2}+\beta_{1}\sigma_{t-1}^{2}$$

Given that the investor sentiment represented by Baidu search keywords may affect stock returns, we add the Baidu Index variable to the above formula (3) to extend the benchmark model. But we need to first determine the lagging term of the Baidu Index. The estimation results show that selecting the lag period 0 can pass the 1% significance test. This is mainly because the Baidu Index search terms are based on days and are reflected in real-time. In other words, the investor sentiment reflected in the Baidu Index may be reflected in the stock market returns of the day. To this end, we establish the following extended GARCH model including the Baidu Index:

(4) $$\sigma^{2}=\omega +\alpha_{1}u_{t-1}^{2}+\beta_{1}\sigma_{t-1}^{2}+\gamma LnBaiduI\text{n}dex$$

Based on the extended model of Equation (4), we use the one-step forward method to predict the return of epidemic stocks. The entire sample period covers March 4, 2020 to March 9, 2021. We first use the time series data from March 4, 2020 to September 6, 2020 to estimate the model, and then we use the above model to predict the variance on September 7, 2020. We continue to repeat the above steps, using the one-step forward method, to predict the variance of the entire sample period. At the end of the sample period, we can use the prediction error to judge the prediction accuracy of the model.

**Figure 1:**
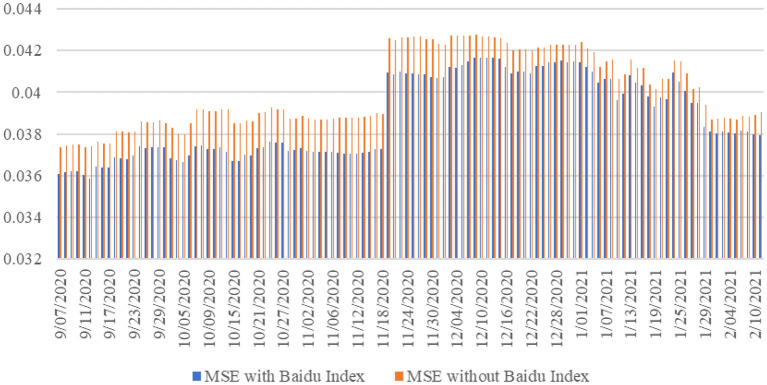
MSE of the benchmark and extended models.

The prediction error value of a model is the difference between the actual model and the conditional variance of the predicted value. The forecast results are shown in Figure 1. Overall, the extended model with the Baidu index has smaller prediction errors. Our research also finds that in periods of high volatility, neither the benchmark model nor the extended model with the Baidu index can explain its variance well. The main reason is that in the period of market turbulence, investors tend to search for information and buy or sell stocks, which leads to substantial turbulence in stock returns.

To accurately calculate the degree of improvement in the prediction accuracy of the extended model relative to the benchmark model, we calculate the root mean square error of the two models. The calculation results show that, compared with the benchmark model, the extended model with the Baidu index reduces its root mean square error by an average of 3.16%. We also divide the entire sample period into a period of high volatility and a period of low volatility. High volatility refers to the period when the conditional variance exceeds the mean. Low volatility refers to periods that are below average. The results of the study show that during periods of high volatility, the number of searches on the Baidu index is also higher. This shows that in turbulent phases of the market, investors increase their information searches to assist in decision-making to avoid risks. This sentiment is directly reflected in the increase in transaction volume. In addition, the calculation results show that the degree of MSE decline in high volatility periods is more significant. Overall, regardless of the period, the extended model including the Baidu Index has greater prediction accuracy than the benchmark model. This shows that the extended model that includes the Baidu index is more suitable for predicting the returns of epidemic stocks. In other words, given the real-time nature of investor retrieval data, investor sentiment can be reflected in epidemic stocks in a timely manner. This has a shorter time lag than the previous official release of data with a significant lagging period on stock prices. Regardless of whether we consider investors or market regulators, including real-time investor sentiment into the prediction model will help improve investment returns or predict the direction of the stock market in real time. Incorporating diversified big data into stock and financial market analysis has become a standard practice.

## Conclusions

In stock investment, although professional investors will monitor the leading index, this approach is largely inaccessible to retail investors due to cost and ability. In the raging situation of the COVID-19 epidemic, once a drastic change in the epidemic is discovered, investors may use search engines such as Google and Baidu as a source of information to assist in investment decisions. Epidemic data obtained by using the Baidu index can well represent investor sentiment and concern about epidemic stocks. After searching for relevant information on stocks related to the epidemic, investors may take investment actions immediately or the next day to affect stock returns.

In this paper, we use Baidu index data to measure investors' interest in searching for stock information about the epidemic. We find that during periods of severe market turbulence, investors' attention to the stock market increases substantially. To avoid risks, investors tend to increase the trading volume of stocks, which leads to large fluctuations in stock prices. By incorporating the Baidu index into the extended GARCH model, we find that investor sentiment is reflected in the stock returns of the epidemic in a timely manner. Especially in periods of market turbulence, the model including the Baidu index can significantly improve forecasts. Therefore, investor sentiment reflected in search engine data constitutes a valuable source of information for predicting future volatility. Such real-time information can improve investor returns and help regulators to monitor the trends of the epidemic in real time. This provides information and decision-making reference points for controlling the epidemic and judging the demand for epidemic prevention materials. Under the COVID-19 pandemic, we can also predict the outbreak of the epidemic and the demand for medical supplies in advance by monitoring investors to search for keywords, and provide a scientific basis for the formulation of a vaccine plan.

## Data Availability Statement

Publicly available datasets were analyzed in this study. This data can be found here: index.baidu.com.

## Author Contributions

BJ: writing—original draft. HZ: writing—review. JZ: investigation and design. CY: draft writing and software. RS: editing. All authors contributed to the article and approved the submitted version.

## Conflict of Interest

The authors declare that the research was conducted in the absence of any commercial or financial relationships that could be construed as a potential conflict of interest.
